# Modeling, Degradation Study, Failures Diagnosis and Faulty Operating Management of Electrolyzers

**DOI:** 10.3390/membranes12121195

**Published:** 2022-11-27

**Authors:** Damien Guilbert, Georgios Papakonstantinou

**Affiliations:** 1 Group of Research in Electrical Engineering of Nancy (GREEN), Université de Lorraine, F-54000 Nancy, France; 2 Max Planck Institute for Dynamics of Complex Technical Systems, Process Systems Engineering, Sandtorstr.1, D-39106 Magdeburg, Germany

Hydrogen is considered an effective solution to deliver or store energy. It is characterized by high energy density (120 MJ/kg) compared to classical energy storage devices (e.g., batteries). In the energy delivery process, hydrogen can supply fuel cells (FCs) for electricity, power, or heat production; in the energy storage process, it can be generated using techniques such as fossil fuel reforming, direct solar water splitting, or water electrolysis. Among these techniques, water electrolysis is an attractive option due to the impact of its low carbon emissions. In this electrochemical process, an electrolyzer (EL) is used to split de-ionized, pure, or distilled water into hydrogen and oxygen, which is fed by low-carbon power generation sources such as renewable energy sources (RES) and nuclear sources [1,2].

Four main electrolyzer technologies have been reported in the literature, depending on the required electrolyte material and the ionic species they diffuse: alkaline, proton exchange membrane (PEM), solid oxide (SO), and anion exchange membrane (AEM) technologies. Due to its operational characteristics, PEM technology is the most promising and attractive solution to cope with the intermittency of RES, since it can quickly respond to variations in the injected power, enabling energy absorbance during fast dynamics [3]. However, under dynamic operation conditions, the membrane may be subjected to degradation [4]. Moreover, the operating conditions (pressure, temperature, current density) [5,6,7] and power electronics may also cause degradation in the membrane [8,9,10].

Furthermore, the reliable operation of ELs is a challenging task due to the presence of several types of failures that can cause premature equipment replacement and operation shutdowns; for example, in ELs, the membrane electrode assembly (MEA) can be subjected to membrane pinhole formation, internal gas leakage, cell drying, and poisoning of the catalyst areas [11]. Since these systems are exposed to several types of failures and degradation according to their operating conditions, modeling, degradation study, failure diagnosis, and faulty operation management must be considered. Only by enhancing EL technologies can hydrogen be introduced as a safe and sustainable energy carrier.

For this Special Issue, we sought original high-quality papers and review articles focused on hydrogen technologies related to their modeling, degradation, failure diagnosis, and faulty operation management.

Topics of interest include, but are not limited to:

- Membrane electrode assembly modeling of electrolyzers;
- Impacts of dynamic operating conditions on the materials and components degradation of electrolyzers;
- Influence of the operating conditions (temperature, pressure, current density) and power electronics on the degradation of electrolyzers;
- Failure mechanisms of electrolyzers;
- Development of failure-diagnosis methods;
- Development of faulty operation management to enhance the performance and lifetime of the system.

As a result of this Special Issue proposal, five research papers [12,13,14,15,16] from four different countries have been published. Researchers at academic institutions and the French National Centre for Scientific Research (CNRS) from France [12,13,15], Mexico [15], Germany [14], and Taiwan [16] contributed to this work. It is important to point out that the research reported in Ref. [12] was carried out in collaboration with the French company Air Liquide, an expert in the design, construction, and operation of low-carbon hydrogen production plants relying on water electrolyzers.

Most of the published papers investigated PEM electrolyzers [12,14,15,16] which confirms the growing interest in this technology to generate hydrogen from RES. Moreover, all the published papers reported experiments performed on electrolyzers that demonstrate the relevance of the papers and the significance of using experimental data for investigation purposes. Hence, the published papers reported on new contributions compared to the current literature, including an investigation of the effects of current ripple from power electronics on the performance of PEM electrolyzers [12]; the use of X-ray micro-computed tomography to examine the 3D microstructure of the anode-electrolytes of electrolyzers [13]; the development of a mathematical model to evaluate the cell polarization, current density distribution and water flow paths inside a cell under low stoichiometry conditions [14]; the analysis of the decrease in the open-circuit voltage of the PEM electrolyzer during shutdown operation and its modeling [15]; and the application of micro-electro-mechanical systems to monitor, in real-time, the state-of-health of a PEM electrolyzer [16].

Starting from these new contributions, some challenging issues remain, such as the modeling of electrolyzer behaviors during dynamic operating conditions (with the use of RES); the investigation of electrolyzers during the dynamic operation, shutdown, start/stop conditions, and those fed by low and high-frequency current ripple (in terms of amplitude and frequency); and the development of fault-diagnosis methods and state-of-health management of electrolyzer systems.

## References

[1] Maestre V., Ortiz A., Ortiz I. (2021). Challenges and prospects of renewable hydrogen-based strategies for full decarbonization of stationary power applications. Renew. Sustain. Energy Rev..

[2] Yue M., Lambert H., Pahon E., Roche R., Jemei S., Hissel D. (2021). Hydrogen energy systems: A critical review of technologies, applications, trends and challenges. Renew. Sustain. Energy Rev..

[3] Guilbert D., Vitale G. (2021). Hydrogen as a Clean and Sustainable Energy Vector for Global Transition from Fossil-Based to Zero-Carbon. Clean Technol..

[4] Papakonstantinou G., Algara-Siller G., Teschner D., Vidaković-Koch T., Schlögl R., Sundmacher K. (2020). Degradation study of a proton exchange membrane water electrolyzer under dynamic operation conditions. Appl. Energy.

[5] Di Blasi A., Andaloro L., Siracusano S., Briguglio N., Brunaccini G., Stassi A., Aricò A., Antonucci V. (2013). Evaluation of materials and components degradation of a PEM electrolyzer for marine applications. Int. J. Hydrogen Energy.

[6] Chandesris M., Médeau V., Guillet N., Chelghoum S., Thoby D., Fouda-Onana F. (2015). Membrane degradation in PEM water electrolyzer: Numerical modeling and experimental evidence of the influence of temperature and current density. Int. J. Hydrogen Energy.

[7] Sun S., Shao Z., Yu H., Li G., Yi B. (2014). Investigations on degradation of the long-term proton exchange membrane water electrolysis stack. J. Power Sources.

[8] Buitendach H., Gouws R., Martinson C., Minnaar C., Bessarabov D. (2021). Effect of a ripple current on the efficiency of a PEM electrolyser. Results Eng..

[9] Speckmann F., Bintz S., Birke K. (2019). Influence of rectifiers on the energy demand and gas quality of alkaline electrolysis systems in dynamic operation. Appl. Energy.

[10] Koponen J., Ruuskanen V., Kosonen A., Niemela M., Ahola J. (2019). Effect of Converter Topology on the Specific Energy Consumption of Alkaline Water Electrolyzers. IEEE Trans. Power Electron..

[11] Millet P., Ranjbari A., de Guglielmo F., Grigoriev S., Auprêtre F. (2012). Cell failure mechanisms in PEM water electrolyzers. Int. J. Hydrogen Energy.

[12] Parache F., Schneider H., Turpin C., Richet N., Debellemanière O., Bru É., Thieu A.T., Bertail C., Marot C. (2022). Impact of Power Converter Current Ripple on the Degradation of PEM Electrolyzer Performances. Membranes.

[13] Lescure V., Gelin M., François M., Arab Pour Yazdi M., Briois P., Demoisson F., Combemale L., Valton S., Caboche G. (2022). X-ray Micro-Computed Tomography: A Powerful Device to Analyze the 3D Microstructure of Anode-Electrolyte in BaZr0.8Y0.2O3 Protonic Ceramic Electrochemical Cells and the Reduction Behavior. Membranes.

[14] Immerz C., Bensmann B., Hanke-Rauschenbach R. (2021). Model-Based Analysis of Low Stoichiometry Operation in Proton Exchange Membrane Water Electrolysis. Membranes.

[15] Hernández-Gómez Á., Ramirez V., Guilbert D., Saldivar B. (2021). Self-Discharge of a Proton Exchange Membrane Electrolyzer: Investigation for Modeling Purposes. Membranes.

[16] Lee C.-Y., Chen C.-H., Jung G.-B., Zheng Y.-X., Liu Y.-C. (2021). PEMWE with Internal Real-Time Microscopic Monitoring Function. Membranes.

